# Milk ladder: Who? When? How? Where? with the lowest risk of reaction

**DOI:** 10.3389/falgy.2024.1516774

**Published:** 2024-12-06

**Authors:** Betul Buyuktiryaki, Ozge Soyer, Duygu Yazici, Gulbin Bingol, Ceren Can, Hikmet Tekin Nacaroglu, Aysen Bingol, Ebru Arik Yilmaz, Metin Aydogan, Cansin Sackesen

**Affiliations:** ^1^ Division of Pediatric Allergy, Koc University School of Medicine, Istanbul, Türkiye; ^2^ Division of Pediatric Allergy, Hacettepe University School of Medicine, Ankara, Türkiye; ^3^ Research Center for Translational Medicine, Graduate School of Health Sciences, Koc University, Istanbul, Türkiye; ^4^ Swiss Institute of Allergy and Asthma Research (SIAF), Davos, Switzerland; ^5^ Division of Pediatric Allergy, Acıbadem Mehmet Ali Aydınlar University School of Medicine, Istanbul, Türkiye; ^6^ Division of Pediatric Allergy, Bakirkoy Dr. Sadi Konuk Training and Research Hospital, Health Sciences University, Istanbul, Türkiye; ^7^ Division of Pediatric Allergy, Medipol University School of Medicine, Istanbul, Türkiye; ^8^ Division of Pediatric Allergy and Immunology, Akdeniz University School of Medicine, Antalya, Türkiye; ^9^ Division of Pediatric Allergy and Immunology, Pamukkale University School of Medicine, Denizli, Türkiye; ^10^ Division of Pediatric Allergy and Immunology, Kocaeli University School of Medicine, Kocaeli, Türkiye

**Keywords:** baked milk, cow's milk protein allergy, food allergy, food ladder, immunotherapy, nutrition, tolerance induction, treatment

## Abstract

The milk ladder (ML) approach, which is the gradual reintroduction of the milk allergen from the least allergenic forms to the most allergenic forms into the diet of the patients, has been utilized mostly in non-IgE-mediated but in some countries also in IgE-mediated-CMPA due to its possible benefits which include nutrition, quality of life and tolerance induction. Despite increasing interest, so far, there is no guideline on ML; thus, the use of this approach shows discrepancies among healthcare professionals as many factors such as dietary habits, patient history, test results, workload, and facilities of the hospitals, the anxiety of the parents/patients may affect the decision on how, when, where and whom to use ML. Here, we reviewed current data on implementing the ML, suggested a 4-step ML including receipts and amounts, and shared our experience on optimal patient selection, appropriate time and steps for initiating ML, and time intervals between the steps targeting the lowest risk of reaction. We also added the newly developed twice-baked biscotti cake to the ML. We presented the analyses of this product, showing its low allergenicity compared to conventional cake, which provides a safer introduction of milk into the diet.

## 1 Introduction

Food allergy is a global healthcare concern that poses a significant burden on physical and psychological health (1). Epidemiologic data reveal that cow's milk (CM) is among the most common food allergens, particularly in infants and young children, with prevalence rates estimated in the range of 2% and 3% when both IgE and non-IgE mediated reactions are considered (2).

Despite the traditional knowledge that cow's milk protein allergy (CMPA) has a favorable prognosis and resolves in pre-school ages, previous studies reported that IgE-mediated CMPA might persist into adolescence or adulthood (3, 4). Compared to IgE-mediated allergy, studies have shown that tolerance develops at earlier ages in non-IgE-mediated food allergies (FA) (5).

In a promising study in 2008, Nowak-Wegrzyn et al. (6) reported that 75% of children with CMPA could tolerate baked forms of milk allergen while still being allergic to fresh milk. Sackesen et al. (7) conducted a study including 89 children with IgE-mediated CMPA; while 18% were reactive even to baked milk (BM), 82% were tolerant to BM (cake), 46% were tolerant to baked and fermented milk products (yogurt, cheese), and 36% of children could consume baked & fermented milk products and fresh milk. Tolerance to BM could be explained by the effects of thermal processing on milk allergens and the food matrix, resulting in a decrease in allergenicity (8). Additionally, interactions with other ingredients such as fat, carbohydrates, and other proteins called food matrix contribute to tolerance to BM by limiting the accessibility of peptides to the immune system (9). Regarding immunologic changes, introducing BM into the diet is associated with increasing serum casein IgG4 antibody levels and decreasing skin prick test (SPT) wheal-size (6). Kim et al. evaluated the long-term effect of introducing BM and changes in immunologic parameters in 88 children (median age 6 years) (10). A significant increase in casein IgG4 levels and a decrease in casein IgE and β-lactoglobulin IgE values were found over a median of 37 months in the BM-tolerant group compared to the BM-reactive group. Moreover, for the patients who incorporated the BM diet, tolerance was 16 times more likely than in the comparison group (10). Based on these observations, management of CMPA has shifted from strict avoidance toward a proactive approach by introducing BM into the diet for tolerance induction, preventing the harmful effect of long-term diets, and increasing the quality of life (11). For this purpose, a milk ladder (ML) is a good alternative for patients with mild-to-moderate CMPA. Additionally, this approach will reduce the use of health care services and increase the diet diversity of the patients.

## 2 Milk ladder: from past to present

The ML is defined as the gradual stepwise reintroduction of the milk allergen from the least allergenic forms, progressing to the most allergenic forms of milk into the diet of the patient (12). The first ML was designed in 2013 by UK researchers who published a paper on diagnosing and managing non-IgE-mediated CMPA in primary care (13). For mild-to-moderate non-IgE mediated CMPA, this “Milk Allergy in Primary Care (MAP)” guideline provided information about a 12-step ML for home reintroduction and the commercially available options and homemade recipes for each step. In 2014, the British Society for Allergy and Clinical Immunology (BSACI) published a guideline for diagnosing and managing CMPA and suggested ML as a treatment option for appropriate patients for both IgE and non-IgE-mediated FA (14). A web-based survey study conducted to evaluate the use of BM challenges and ML in clinical practice by healthcare professionals (HCP) across the world reported that 68% of HCPs use ML in non-IgE mediated FA, 60% use ML also in IgE-mediated FA to determine the children able to tolerate BM-containing foods (15). Since the utilization of the ML was observed, an international interpretation of MAP guideline offering a 6-step ML approach with more healthy recipes was published in 2017 to meet the requirement of HCPs not only in the UK but also in other national healthcare systems in the world (16). Further, Canadian researchers developed Canadian food ladders for pre-school-aged children with mild IgE-mediated FA consisting of 4 steps, including foods that are more acceptable to Canadian households (17). As cultural differences may affect ML use in daily practice, it has also been modified based on the local eating habits in Greece, Spain, Germany, and India (18–21). Recently, World Allergy Organization (WAO) Diagnosis and Rationale for Action against Cow's Milk Allergy (DRACMA) Guideline has been published, including recommendations regarding reintroducing CM as a ML approach in patients with CMPA (22).

In recent years, we have started to use ML in our daily practice to determine clinical reactivity or tolerance of the patients or to accelerate tolerance induction in patients who cannot consume fresh milk but tolerate BM. However, some fatal cases due to the anaphylactic reactions after baked milk pose questions about the usage by physicians (23). In addition, Galletta et al. reported that the anaphylactic reactions during the OIT treatment led to the discontinuation of the treatment by the parents and had a negative impact on treatment adherence (24). Of note, Chua et al. have recently made the very insightful statement that the rate of anaphylaxis in ML programs is no higher than with more conventional OIT or even milk allergy itself (25).

Since this approach is adopted by many practitioners in many countries, in this narrative review, our expert group reviewed the most recent evidence on ML, intending to suggest recommendations for commonly encountered questions and concerns in clinical practice.

## 3 Who is the optimal candidate for the milk ladder?

Children with CMPA are clinically and immunologically heterogeneous and present in different clinical spectrums (6). Older children with severe phenotypes cannot consume even baked forms of milk; thus, the ML can be a good choice for children having mild and moderate forms of the disease, but data regarding young children and infants with anaphylaxis is lacking.

**Figure 1:**
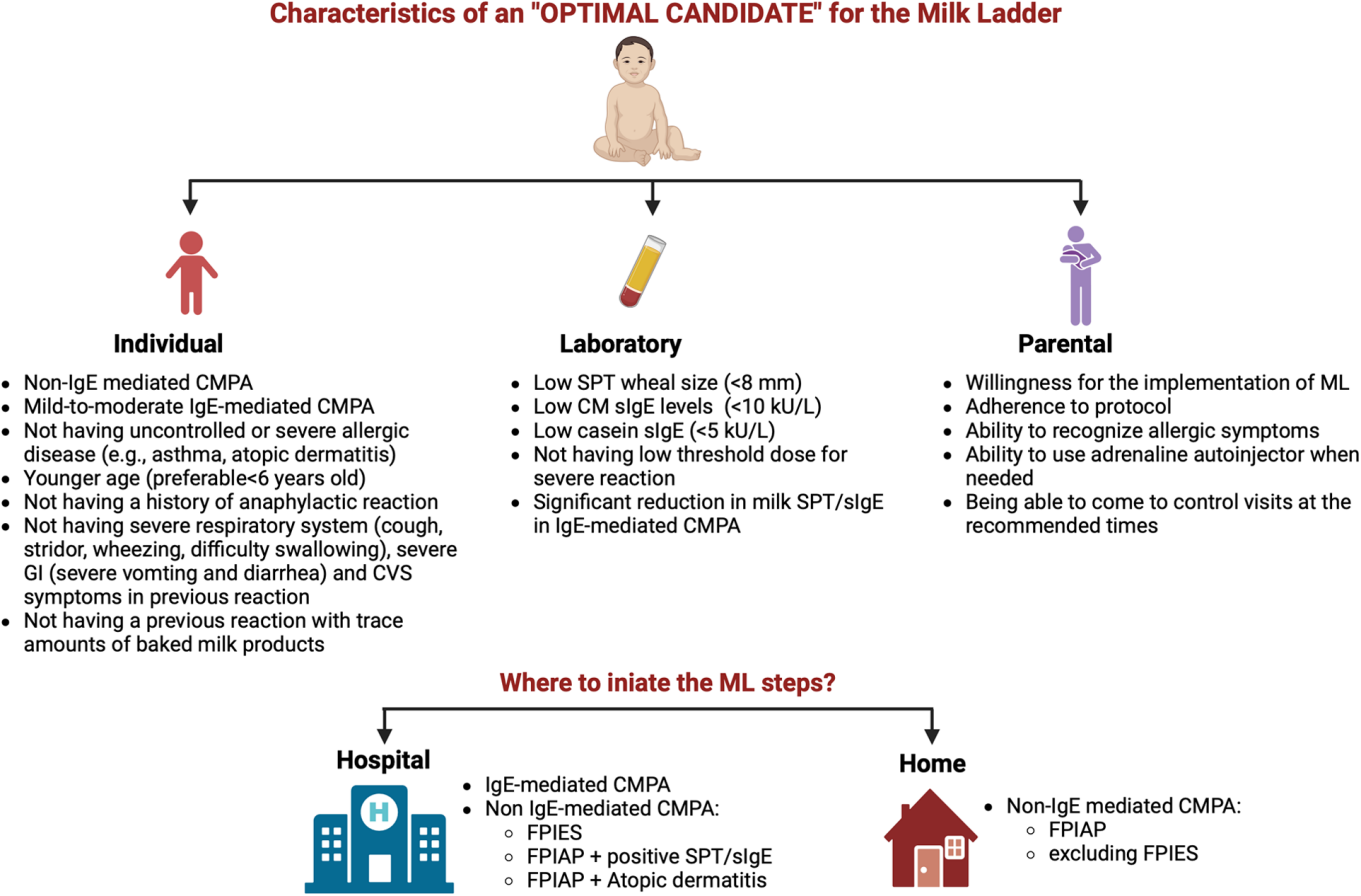
Characteristics of an optimal candidate and the appropriate setting for initiating the milk ladder (CM, cow's milk; CMPA, cow's milk protein allergy; CVS, cardiovascular; FPIAP, food protein-induced allergic proctocolitis; FPIES, food protein-induced enterocolitis; GI, gastrointestinal; ML, milk ladder; SPT, skin prick test; sIgE, specific IgE) (created in https://BioRender.com).

Deciding on the optimal candidate is an important determinant for a safe and successful ML (Figure 1). While ML is accepted as a safer form of reintroducing milk allergen into the diet compared to oral immunotherapy (OIT), the tragic death of 9 years of a girl in Canada who was consuming muffins routinely and under baked milk oral immunotherapy treatment emphasized the importance of patient selection once again (23, 25).

Previous studies that reported successful results with food ladders included younger children without a history of anaphylaxis who are likelier to outgrow their FA. In an ML study, Ball et al. (26) included 86 patients with IgE-mediated CMPA with a median age of 13 months (8–33 months) but excluded the patients who had a history of reaction with trace amounts of BM ingestion or had a reaction involving the respiratory or cardiovascular system, had recurrent wheezing, had SPT > 8 mm. Of the patients, 68 (79.1%) could tolerate all dairy products by 2-year follow-up, and no patient experienced anaphylaxis during the study period.

Severe reactions are mostly associated with high levels of milk and casein sIgE, SPT wheal diameter, low threshold dose, and history of anaphylaxis; therefore, a patient with one of these criteria would not be an optimal candidate for the ML (12). BSACI recommended the home reintroduction of BM in patients with mild symptoms (e.g., cutaneous reactions), no reaction to milk in the last 6 months, and a significant reduction in milk SPT/sIgE in IgE-mediated FA (14). In IgE-mediated CMPA, the WAO-DRACMA guideline recommends initiating ML in patients under 3 years old with an SPT < 8 mm, usually under physician supervision in a medical setting (22).

As to accompanying allergic diseases, patients with uncontrolled asthma or uncontrolled atopic dermatitis (AD) are not suitable for ML (25), but these patients can be re-evaluated after allergic diseases are taken under control. Adherence of the patients and parents is another issue that should be considered. Before starting ML, an interview explaining the details of the protocol should be done with the parents, and the benefits along with the risks of the treatment process need to be discussed. Parents must adhere to the regular consumption of the foods in the steps and must be competent in treating allergic reactions when occurs (Figure 1).

In respect of age, young children were reported to have fewer reactions involving lower respiratory, cardiovascular, and neurologic symptoms during oral food challenge (OFC) tests compared to older age groups (27). Likewise, older age was found as a risk factor for severe reactions during double-blind placebo-controlled food challenges (DBPCFC) in a retrospective study (28). So far, studies in older children and adolescents are limited; thus, the efficacy and safety of the ML in this age group need to be further investigated due to having a more persistent disease course.

In agreement with available evidence, a food ladder safety checklist has been published in a recent paper. Chua et al. (25) proposed the checklist to assist with patient selection as 4A's, including Age, Asthma, history of Anaphylaxis, and Adherence. Based on this checklist, patients who are older than 6 years of age, who have severe or uncontrolled asthma, have a history of a severe reaction to tiny amounts of food, especially in baked products, and are unable to commit to daily doses of ladder might not be a good fit for food ladder (25).

Even if the ML is preferred in patients without severe phenotype, there is still a risk of developing anaphylaxis (12). Additionally, the presence of co-factors such as physical exercise, drugs (e.g., non-steroid anti-inflammatory drugs), and acute infections, body temperature changes, menstruation, some other systemic conditions such as cardiovascular disease and mastocytosis may elicit an allergic reaction with lower doses of allergen and should be kept in mind while preparing a home protocol, and parents should be informed (29–31).

A subset of patients with eosinophilic esophagitis has also been shown to tolerate BM products, however, more studies are required on this subject (32). For non-IgE-mediated CMPA, the data on food protein-induced enterocolitis syndrome (FPIES) is lacking. On the other side, home re-introduction is commonly implemented in patients with non-IgE-mediated gastrointestinal diseases (excluding FPIES) by HCPs in clinical practice, and an ML is a safe approach for these patients (33). Of note, children with FPIAP may have accompanying IgE sensitization to CM and/or AD; hence, parents need to be informed about the symptoms of early and delayed type allergic reactions (5).

## 4 When to initiate the milk ladder?

So far, there is no absolute agreement on the appropriate age for the ML. For this reason, the time to start the ML is usually determined by the physicians on an individual basis, and different approaches, even between different clinic centers in the same country, are seen.

In the UK, the IMAP ML has been commonly started from 1 year of age (13, 16). Although the authors recommended the use of the ML in non-IgE-mediated FA, in clinical practice, it is also initiated in IgE-mediated FA, whether at home or in a hospital setting depending on the decision of the physician (15, 16). The Canadian Food Ladders were developed to use in “preschool-aged children” with mild IgE-mediated reactions to CM. This recommendation is based on the safety data of OIT studies in which OIT has been shown to be more effective in young children and observation of less severe reactions in this age group (34, 35). Differently, in Ireland, due to limited accessibility to pediatric allergists, the MAP ML is initiated at home for both IgE and non-IgE-mediated CMPA when the infants are weaned onto solid foods (36). In a recent study in Ireland by d'Art et al. (36), safety and efficiency of initiating ML at diagnosis following a supervised single dose of fresh milk at the ED05 at hospital have been reported in patients with IgE-mediated CMPA under 1 year of age. The authors suggested that a supervised single dose of fresh milk at the ED05 significantly accelerates the progress at ML, probably by giving parents the confidence to proceed.

A cut-off CM sIgE value for initiating ML has not been determined yet, but studies regarding baked milk challenges may help clinicians if they would prefer to administer ML approach in patients who are more likely to tolerate baked milk. In the study of Nowak et al. (6), among the 100 children with CMPA who underwent the heated milk challenge, none of the patients with negative serum CM-sIgE levels (<0.35 kUA/L) or SPT mean wheal diameters <5 mm showed reactivity to heated milk, on the other side 85% of the children with CM-sIgE > 35 kUA/L had allergic reactions during the heated milk challenges. Later, Caubet et al. (37) evaluated 225 children prospectively, including the 100 children in the previous study. The authors suggested a cut-off of 5 kU/L casein-sIgE (74% sensitivity, 77% specificity, 89% NPV, and 54% PPV) and a cut-off of 10 kU/L CM-sIgE (62% sensitivity, 85% specificity, 86% NPV, and 60% PPV) for passing a BM challenge. Patients with a level of casein-sIgE higher than 20.2 kU/L were found more likely to be reactive to BM and avoidance of BM products is recommended. Bartnikas et al. (38) retrospectively analyzed the outcomes of OFC tests with BM in 35 children. All children with a CM-SPT < 7 mm passed the BM challenge, moreover, patients with casein-SPT < 9 mm were found 92.3% likely to tolerate BM. None of the children with casein-SPT > 15 mm, casein-sIgE > 10.3 kU/L and CM-sIgE > 20.6 kU/L could tolerate BM.

In untreated IgE-mediated CMPA, intervals for re-evaluation are recommended every 6–12 months, and patients with a decrease of 50% or more in CM-sIgE levels have a more favorable prognosis for the development of tolerance (39, 40).

Physicians might prefer using the ML approach to determine patients' tolerance rather than performing OFCs with fresh milk. For non-IgE-mediated FA, a CM-free diet is recommended until 9–12 months of age and for at least 2–6 months (16). In these patients, reintroduction can be initiated with an ML approach. In a recent study, the challenge of patients with allergic proctocolitis is recommended at 2 monthly intervals. WAO-DRACMA guideline recommends a therapeutic elimination diet for at least 6 months or up to 9–12 months if the OFC confirms the diagnosis of CMA (22).

## 5 What are the foods on each step in the milk ladder?

**Figure 2:**
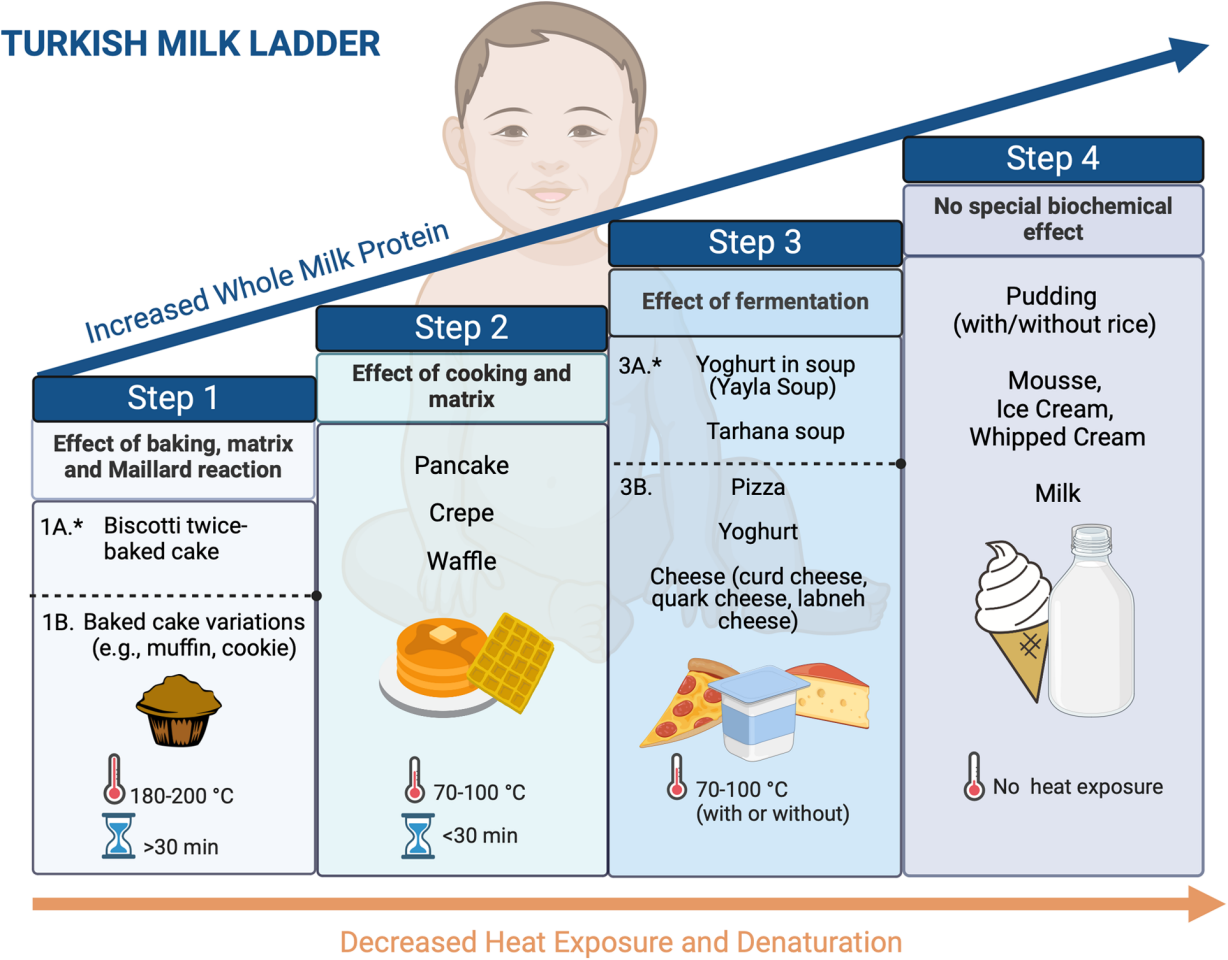
Four-step milk ladder. *1A and 3A products are optional based on the clinician's decision (created in https://BioRender.com).

**Figure 3:**
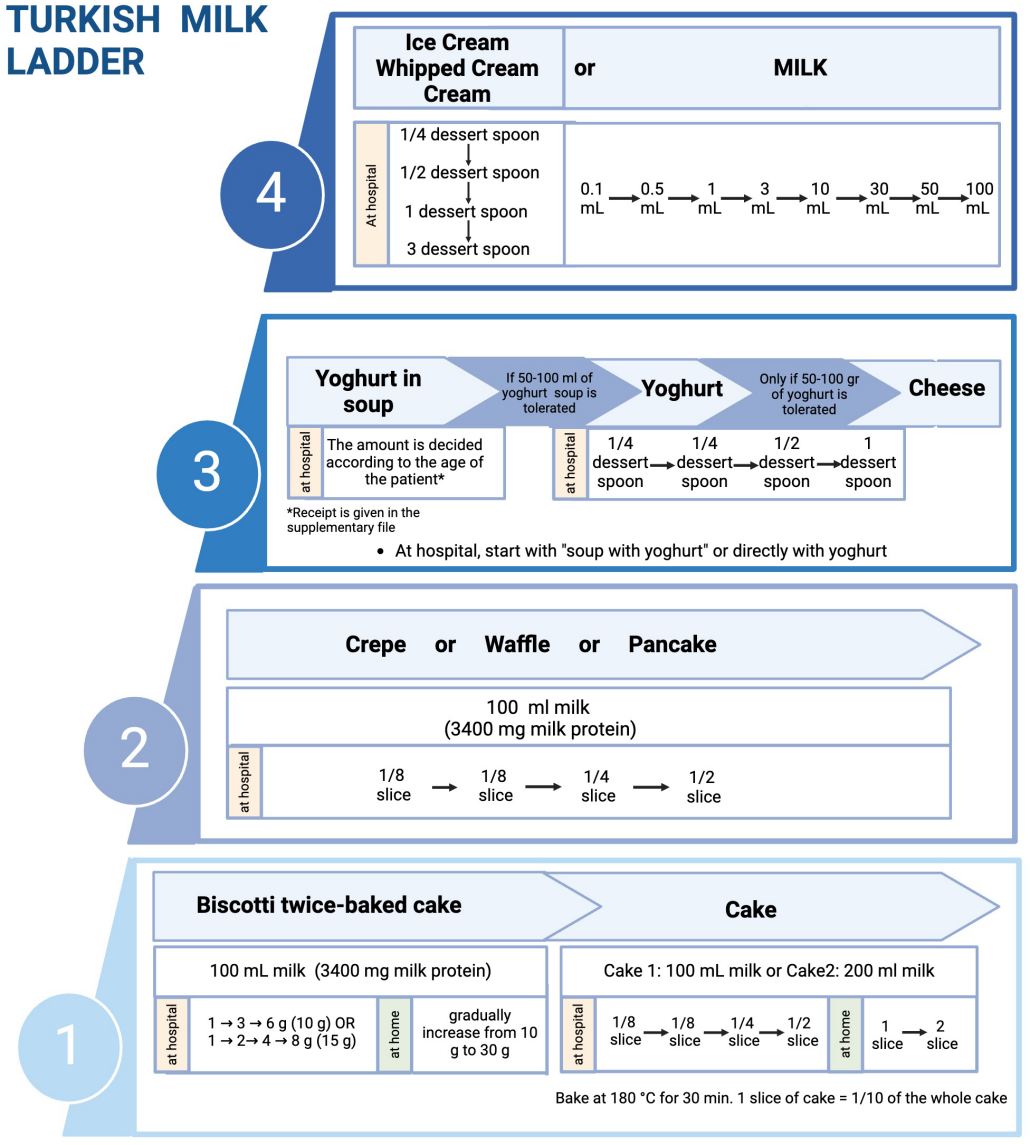
Implementation of milk ladder in clinical practice (created in https://BioRender.com).

Implementing the ML may vary based on the traditional food culture and needs to be adapted for each population. In this review, we propose a 4-step ML including home-prepared products, which we find acceptable to our population and different cultures worldwide (Figures 2, 3) (Recipes for the foods included in the steps are presented in Supplementary Tables S1–S4).

In general, baked milk products such as cakes and muffins are the first foods recommended to be introduced in ML, but some patients may still experience reactions to them. To create a safer product, we developed a new low-allergenic baked milk product named biscotti-twice-baked cake, which showed its low allergenicity compared to the conventional once-baked cake by experiments (41).

The biscotti-twice-baked cake is prepared by combining 100 ml of condensed milk, 125 g of sugar, 250 g of flour, 10 g of baking powder, and 60 ml of vegetable oil to form a conventional dough for a milk cake (Supplementary Table S1). Proceed to chill the dough overnight at a temperature of −20°C. The following day, the mixture undergoes a 30-minute baking process at a temperature of 180°C. Following that, it is let to cool and then cut into slices. The slices are subjected to a second baking procedure maintained at a low temperature between 90°C and 120°C for a period of 3 h (41). To achieve consistent and even distribution of reduced allergenicity across all product parts, including exterior and inside, it is necessary to bake it at a temperature of 90°C for 3 h. Nevertheless, the biscotti can be considered too firm for youngsters to consume. Hence, we employ a manual grinding process using a mortar to produce a powdered form of biscotti that exhibits greater uniformity and facilitates precise measurement in tiny quantities (41). The biscotti powder enables us to accurately measure and distribute the desired quantities of biscotti powder in little cups for a period of 1 month. Both the parents and children reported satisfaction with the biscotti powder. The biscotti-twice-baked cake is similar to the biscuit in the previous ladder, but here we present in-house made product in which how much CM protein in 1 g of powder is known. The recipe of the in-house biscotti-twice-baked cake is simple and easy to prepare. In this way, parents can bake it at home by themselves.

**Figure 4:**
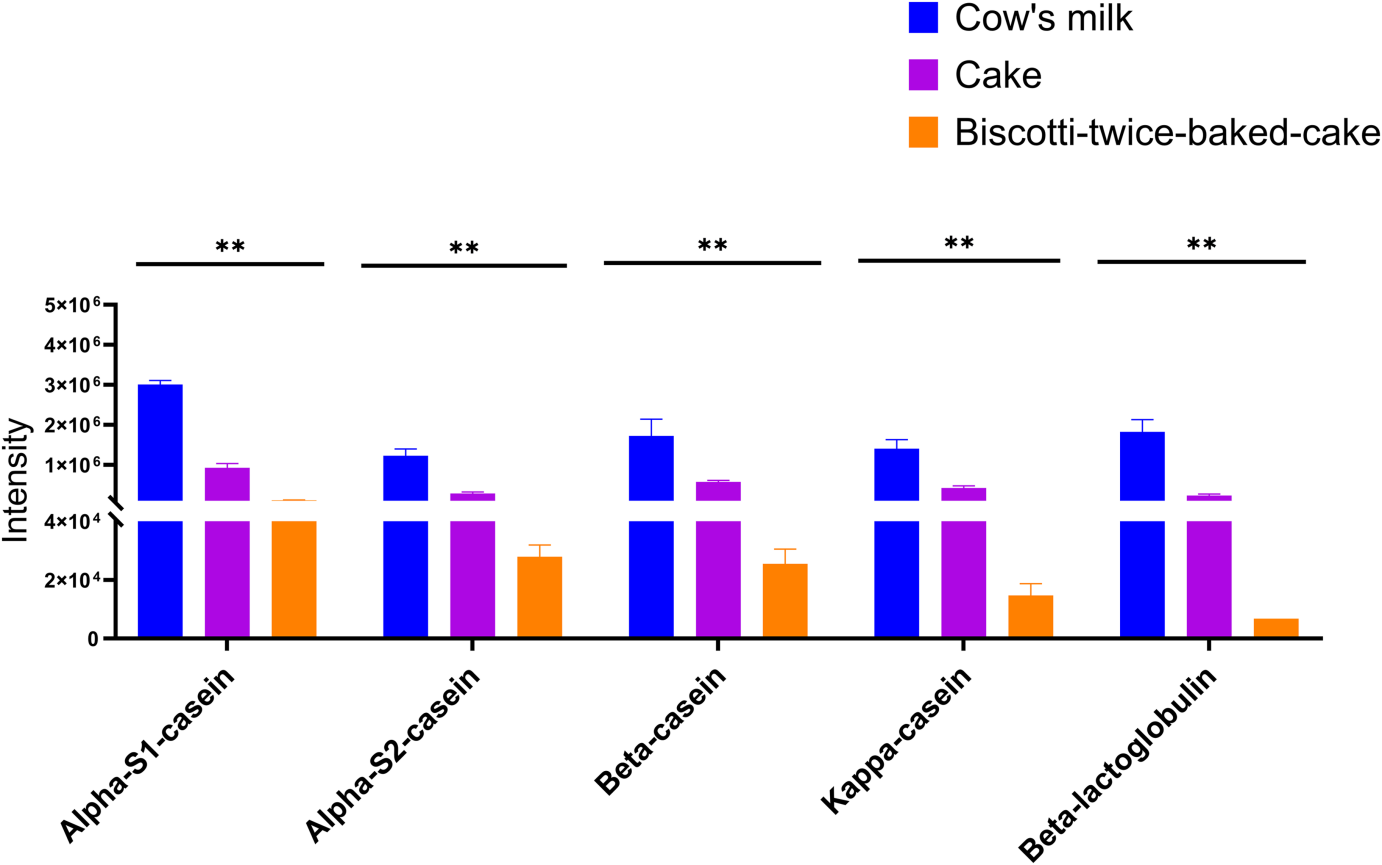
The proteomics analysis by LC-qTOF-MS.

**Figure 5:**
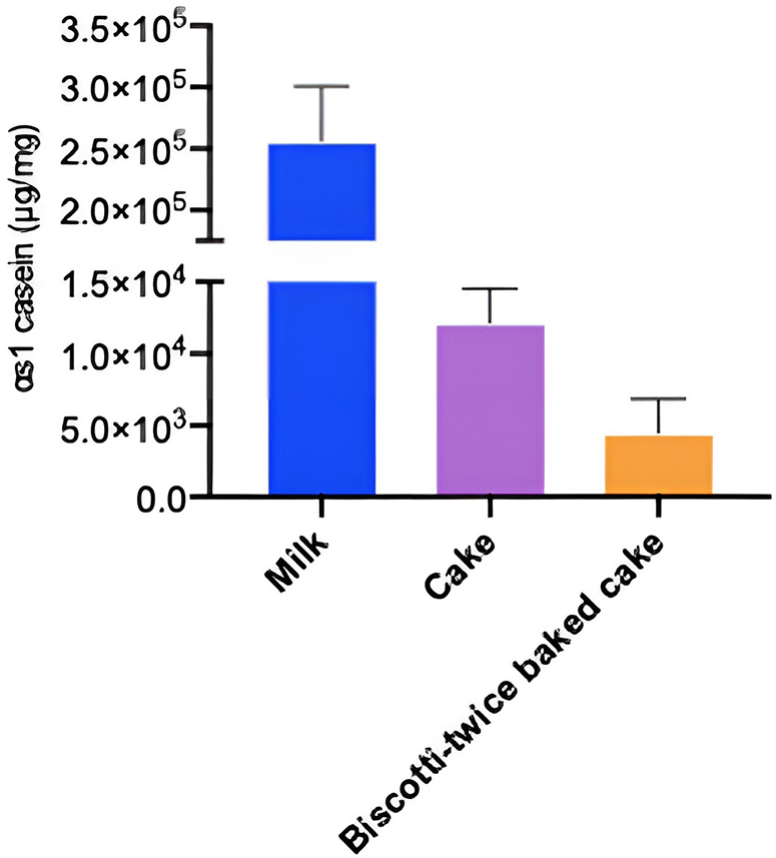
ELISA evaluated αs1 casein concentration for milk, cake and biscotti.

When we investigated the hypo-allergenicity of biscotti-twice-baked cake with the proteomics analysis by LC-qTOF-MS, a decrease in the intensities of each casein fraction and β-lactoglobulin with increasing baking time was observed (Figure 4). Upon comparing the levels of intact protein in CM, cake, and biscotti-twice-baked cake, it was observed that the lowest level was present in biscotti-twice-baked cake, while the highest level was identified in CM (Figure 4). In ELISA, αs1 casein concentration level was lower in biscotti-twice-baked-cake than in conventional cake (Figure 5). The highest level was observed in CM.

Our previous study showed that milk sIgE of milk-allergic patients exhibited incompetently binding to casein bands in biscotti-twice-bake cake compared to conventional cake and pasteurized CM (41). As an additional and optional baked milk product in step 1, clinicians may prefer initiating ML with our new biscotti-twice-baked cake, which is low-allergenic than conventional baked milk products and can be prepared at home. In clinical practice, the ML can be initiated by performing an OFC at the hospital with 10 g (85 mg milk protein) or 15 g (128 mg milk protein) biscotti twice-baked cake and gradually increased to 30 g biscotti at home (Supplementary Table S1). After that, the patient can proceed to the cake step.

Compared to the previous ladders, the Turkish ML introduces yogurt first in soup (Yayla soup) in the fermented milk products step (Figures 2, 3). As part of our traditional cuisine, we incorporate another soup, named tarhana soup, into our meals, which is made with yogurt. Consequently, we have included this soup as part of step 3. If the patient exhibits no allergic reaction to consuming yogurt soup (Yayla soup) or tarhana soup, we proceed to include yogurt and/or cheese into their diet. As an optional step, physicians can prefer to initiate step 3 with yoghurt in soups before directly introducing yoghurt and cheese directly to their patients based on the clinical history and laboratory results.

## 6 Where to initiate the milk ladder? At home or the hospital?

Where to initiate ML steps depends on the type and severity of FA. The determination of risks and benefits guides our decision, not only on the timing of performing OFC but also on the setting of the introduction of food, e.g., home or hospital (42, 43) (Figure 1). At the beginning of the ML performing the initial milk product challenge at each step at the hospital is preferable. If the patient tolerates enough amount of milk product, the gradual increase of the amount at the same step can be continued at home. The details of the Turkish ML shown in Figure 3 show the integration of hospital and home intervention protocols.

### 6.1 Non-IgE-mediated CMPA

#### 6.1.1 Food protein-induced allergic proctocolitis

Dietary elimination of the causative food is recommended for 3–6 months (16, 44). Subsequently, home reintroduction is advised using an ML approach (16, 44, 45). Meyer et al. (33) demonstrated the safety of home food introduction protocols based on a food ladder approach in patients with non-IgE mediated FA not only with milk but also egg, wheat, and soya. AD and/or IgE-mediated FA may coexist with FPIAP (5, 46). Occasionally, infants with FPIAP may develop IgE-mediated FA over time to offending foods (47, 48). SPT and/or serum sIgE testing may be required in infants with AD, a history of immediate onset of allergic symptoms, and after a long period of avoidance before food reintroduction (16, 45).

#### 6.1.2 Food protein-induced enterocolitis syndrome

Our expert group recommends performing OFC under close supervision in a hospital setting where HCPs experienced in treating an acute FPIES reaction are present (42). WAO-DRACMA also recommends initiating ML typically under physician supervision in a medical setting but also suggests that patients with mild symptoms to large amounts of liquid milk might be considered for a very gradual home introduction (22).

There is inconclusive data concerning the setting of the first administration of high-risk foods in infants with FPIES either in the hospital or at home. However, the severity of reaction in FPIES is shown to be dose dependent (49). If the home-based trial is preferred, a slower-graded approach seems safer (50). Cow's milk is reported as a common cause of FPIES (51–53). The feasibility of the ML approach has not been studied in patients with milk FPIES however, some children may only consume BM products (54). Ocak et al. (54) reported that two patients with milk FPIES were able to eat yogurt, but could not tolerate pasteurized milk.

### 6.2 IgE-mediated CMPA

Baked milk challenges have a risk of severe, even fatal, allergic reactions, sometimes requiring more than one adrenaline injection for management (18, 23, 55). Thus, many reports advise the first introduction of BM products under close medical supervision, preferably in a hospital setting (16, 22, 42). On the other side, a retrospective analysis comparing the management strategies of IgE-mediated CMPA in Ireland and Spain reported that primary care is a safe and effective setting to employ the ML (56).

Mehr et al. (55) demonstrated the risk factors for clinical reactivity to BM as asthma, multiple IgE-mediated FA, and a history of CM-related anaphylaxis. Although home-based CM reintroduction has been reported as safe in a recent study excluding children with high risk, currently, our expert group recommends initiating ML at the hospital in patients with IgE-mediated CMPA which is also compatible with WAO-DRACMA guideline (26).

## 7 Dosing strategies in OFC tests of the milk ladder steps

**Figure 6:**
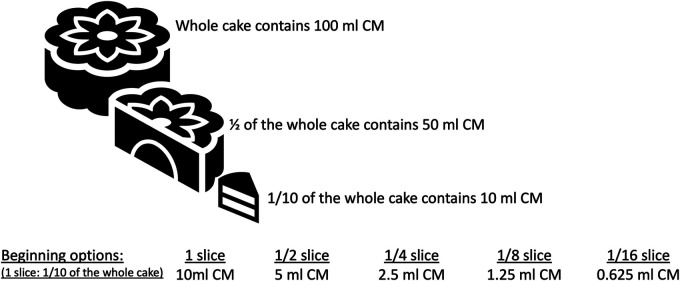
Cow's milk amounts in cake.

In IgE-mediated and, in some cases with non-IgE-mediated CMPA, we initiate each step at hospital setting and perform OFC tests. Different allergy centers have different approaches regarding dose numbers and intervals in OFC (57). Generally, the target number of steps in OFC is 6–8, but in infants, since the total amount is low, the steps of OFC may be reduced to three or four steps, and the interval between steps should be between 15 and 30 min, depending on the risk of a reaction. In recent years, Japanese researchers have recommended dose intervals of 20–60 min to maximize the safety of OFCs (57). The Japanese guidelines for FA state that OFC can be performed with a single dose or two doses in patients with low risk of reaction after food exposure and in 5 divided doses at intervals of 20–60 min in patients with high risk (57–59). The cumulative amount of food in the OFC test should be administered considering the child's age and stomach capacity. The approach which is shown in Figure 6 is feasible by dividing 1 slice of cake (complete cake contains 100 ml CM and 1/10 of the cake is equal to 1 slice and 1 slice contains 10 ml CM (300 mg CM protein) to 4, 8 or 16 according to the risk of reaction. As shown in Figure 3 at step 1 you can perform the challenge with baked milk product, cake, and start with ¼ of the slice and continue 1/4 → 1/2 (1/8 → 1/4 → 1/2 or 1/4 → 3/4) or in favorable higher risk patient start with 1/16 continue 1/16 → 1/8 → 1/4. The amount of the cake or yogurt (cheese) in each step of OFC is up to the physician, considering the age, weight, appetite, and willingness of the child.

## 8 The time interval between milk ladder steps

Although ML has been used in CMPA with increasing frequency in clinical practice for the last 10 years, the data on time between the steps are limited, and there is still no consensus on this issue (12). During the ML, as the patient tolerates the foods in an age-appropriate amount in a step, moving-up to the next step. Before moving up from one step to the next one, patients should be able to consume the food in that step at least 3 days a week without any reaction (36). At each step, a decision should be made for the date of the next step.

### 8.1 By the purpose of the ML

In clinical practice, the ML can be used for two main purposes in patients with CMPA; to determine tolerance or induce tolerance/sustained unresponsiveness (16, 60). If the aim is to determine whether the patient can tolerate the foods in the ML, time intervals between steps can be kept short according to the type and phenotype of CMPA. The short step intervals may be used to determine whether tolerance develops in patients with low-risk IgE-mediated CMPA and FPIAP. However, in cases with IgE-mediated CMPA or FPIES or those requiring tolerance induction, schedules with longer step intervals will be safer and more useful for tolerance induction. For example, Nowak-Wegrzyn et al. conducted a study to compare the effect of two MLs with different dose-escalation intervals on tolerance in patients aged 4–10 years with suspected IgE-mediated CMPA (61). They tested two consecutive steps of the ML on the same day to determine which foods in the ladder the patients could tolerate before the randomization. According to this approach, on the first day, they tested up to two foods from different steps of ML, such as muffins and pizza, 2–3 h apart. If a patient passed both steps without reactions, they performed a challenge test with rice pudding in the next step within 2 weeks. This approach allows a rapid determination of which step in the ML the patient can tolerate. For patients with IgE-mediated CMPAs, the time intervals for consecutive food groups in the different steps of the ML to be challenged should be at least 2 h to observe the reactions accurately (61). However, this approach may not be appropriate for patients with high-risk IgE-mediated CMPA phenotype, and non-IgE mediated CMAs where the reactions occur in the late period. For FPIAP, it will be proper to keep the intervals between steps for 1–2 weeks to observe better the reactions that may develop in the late period (62). Although there is no data for FPIES on this subject, it does not seem appropriate to try two subsequent steps on the same day, considering that the symptoms appear 1–4 h after ingesting the offending food and end within 24 h (63).

Based on data of OIT for FA, if the purpose of the ML is to induce tolerance and change the immunological parameters, the time on each step should be kept longer. This approach may be more appropriate and safer in moderate to high-risk IgE-mediated CMPA and FPIES. The first guide to propose the ML in IgE-mediated CMPA in clinical practice by The BSACI, recommends that the step intervals should be 6 months (14). According to the Canadian ML, when a patient tolerates the age-appropriate portion of food in a step, they move up to the next step after staying at that step for at least 1–3 months (17). In a recent study by Nowak-Wegrzyn et al., the authors compared the effect of escalation steps every 6 or 12 months on progression to tolerance in patients with IgE-mediated CMPA (61). The authors did not find a significant difference in tolerance rates and immunological changes by 36 months between groups with the step intervals of 6 or 12 months.

### 8.2 By the type of CMPA (IgE-mediated or non-IgE mediated)

#### 8.2.1 IgE-mediated CMPA

The reactions usually occur within 2 h in IgE-mediated CMPA. In patients with low-risk IgE-mediated CMPA phenotype, if the aim of the ladder is not to rapidly determine the patient on which step, the time interval between each step can be kept at least 1 week to 1 month to ensure the patient can consume the step foods without any reactions. In cases with high-risk IgE-mediated CMPA phenotype and those intended to induce tolerance, schedules with longer step intervals can be safer. In the ML of BSACI, the first step starts from a small crumb of commercial malt biscuits, reaches up to 1 biscuit per day in weekly increments, and completes in 5 weeks. After the first one, the other steps last 4–6 months (14). Ball et al. suggested that longer time intervals provide sufficient time to ensure the patients can safely tolerate all steps, and the time intervals between steps might be flexible depending on intercurrent infections, the need for dose reduction, and the lifestyle of families (26). In a recent study from Ireland, 82% of patients with suspected IgE-mediated CMPA less than 12 months reached half of the ML at 12 months (36). Although a clear time interval between the steps was not determined in that study, the authors stated that before moving up to the next step, patients should be able to consume the food at that step in an age-appropriate portion at least 3 days a week.

#### 8.2.2 Non-IgE mediated CMPA

The symptoms usually appear 1–72 h after the ingestion of the offending food in non-IgE mediated FA (64). However, this period may be as long as 2 weeks in some patients with FPIAP and chronic FPIES (62, 65). For better observation of the late reactions related to the offending food and to confirm that the patient can consume that food without any reactions, the time interval between the steps is recommended to be 1–2 weeks in cases with FPIAP. However, it may be possible to shorten or lengthen the intervals according to the factors such as the patient's age, clinical findings, and the family's lifestyle. In a recent study, Meyer et al. shortened the duration of each step to 3 days and completed the ML within 2 weeks in patients with non-IgE mediated CMPA except FPIES (33). To date, there is no data regarding the optimal time interval between steps of the ML in FPIES. However, it seems to be safer if the ML step intervals are longer in cases with FPIES.

### 8.3 By the reactions during the ML

The reactions during the ML may affect the time intervals between the steps depending on their severity. In case of mild reactions such as localized pruritus, localized urticaria, or localized flushing, sneezing, or nasal congestion (66) dose reduction can be made in the same step. It may be considered to return to the previous step depending on the reaction severity or the tolerability of the food. Although there is no evidence on how long to stay in the previous step in such a case, it may be kept for at least 1–3 months to move on to the next step more safely (17).

## 9 What are the gaps and further needs?

There is no doubt that beginning ML in children with CMPA encourages the families about the possibility of milk allergy resolution, improves the quality of life, and increases the resolution rate of milk allergy. However, there is no consensus about the conditions which maintain the most possible low number of reactions and prevent severe reactions. Even though a high number of children have benefited from ML in the last decade, it is obvious that physicians should take care when choosing the patient with low risk, deciding about the dose of milk protein containing food from the steps of the ML, optimizing the time intervals to gradually increase the dosages and jumping to the next steps of the ML. We need more studies in infants and young children to identify the instructions of ML. In the near future combining ML and OIT with different strategies to modulate the immune system is promising, but more controlled and well-planned studies are needed to build safe and effective protocols.
