# Corrigendum: Milk ladder: Who? When? How? Where? with the lowest risk of reaction

**DOI:** 10.3389/falgy.2025.1576302

**Published:** 2025-03-14

**Authors:** Betul Buyuktiryaki, Ozge Soyer, Duygu Yazici, Gulbin Bingol, Ceren Can, Hikmet Tekin Nacaroglu, Aysen Bingol, Ebru Arik Yilmaz, Metin Aydogan, Cansin Sackesen

**Affiliations:** ^1^ Division of Pediatric Allergy, Koc University School of Medicine, Istanbul, Türkiye; ^2^ Division of Pediatric Allergy, Hacettepe University School of Medicine, Ankara, Türkiye; ^3^ Research Center for Translational Medicine, Graduate School of Health Sciences, Koc University, Istanbul, Türkiye; ^4^ Swiss Institute of Allergy and Asthma Research (SIAF), Davos, Switzerland; ^5^ Division of Pediatric Allergy, Acıbadem Mehmet Ali Aydınlar University School of Medicine, Istanbul, Türkiye; ^6^ Division of Pediatric Allergy, Bakirkoy Dr. Sadi Konuk Training and Research Hospital, Health Sciences University, Istanbul, Türkiye; ^7^ Division of Pediatric Allergy, Medipol University School of Medicine, Istanbul, Türkiye; ^8^ Division of Pediatric Allergy and Immunology, Akdeniz University School of Medicine, Antalya, Türkiye; ^9^ Division of Pediatric Allergy and Immunology, Pamukkale University School of Medicine, Denizli, Türkiye; ^10^ Division of Pediatric Allergy and Immunology, Kocaeli University School of Medicine, Kocaeli, Türkiye

**Keywords:** baked milk, cow's milk protein allergy, food allergy, food ladder, immunotherapy, nutrition, tolerance induction, treatment

A Corrigendum on Milk ladder: Who? When? How? Where? with the lowest risk of reaction By Buyuktiryaki B, Soyer O, Bingol G, Can C, Nacaroglu HT, Bingol A, Arik Yilmaz E, Aydogan M, Sackesen C. (2024). Front. Allergy. 5:1516774. doi: 10.3389/falgy.2024.1516774

**Error in Author List:**

In the published article, there was an error in the author list and the affiliations, author Duygu Yazici and their affiliations were erroneously excluded. The corrected author list and the affiliations for author Duygu Yazici appears below.

Betul Buyuktiryaki^1^, Ozge Soyer^2^, Duygu Yazici^3,4^, Gulbin Bingol^5^, Ceren Can^6^, Hikmet Tekin Nacaroglu^7^, Aysen Bingol^8^, Ebru Arik Yilmaz^9^, Metin Aydogan^10^, Cansin Sackesen^1*^ on behalf of Turkish National Society of Allergy and Clinical Immunology (TNSACI)

3. Research Center for Translational Medicine, Graduate School of Health Sciences, Koc University, Istanbul, Turkey

4. Swiss Institute of Allergy and Asthma Research (SIAF), Davos, Switzerland

**Error in Figure:**

In the published article, there was an error in Figure 3 as published. The amounts were increased as 1/4→1/4→1/2 →1 slice in step 1 and step 2. The amounts are corrected as 1/8→1/8→1/4 →1/2 slice. The corrected Figure 3 and its caption “Implementation of milk ladder in clinical practice” appear below.

**Figure 3:**
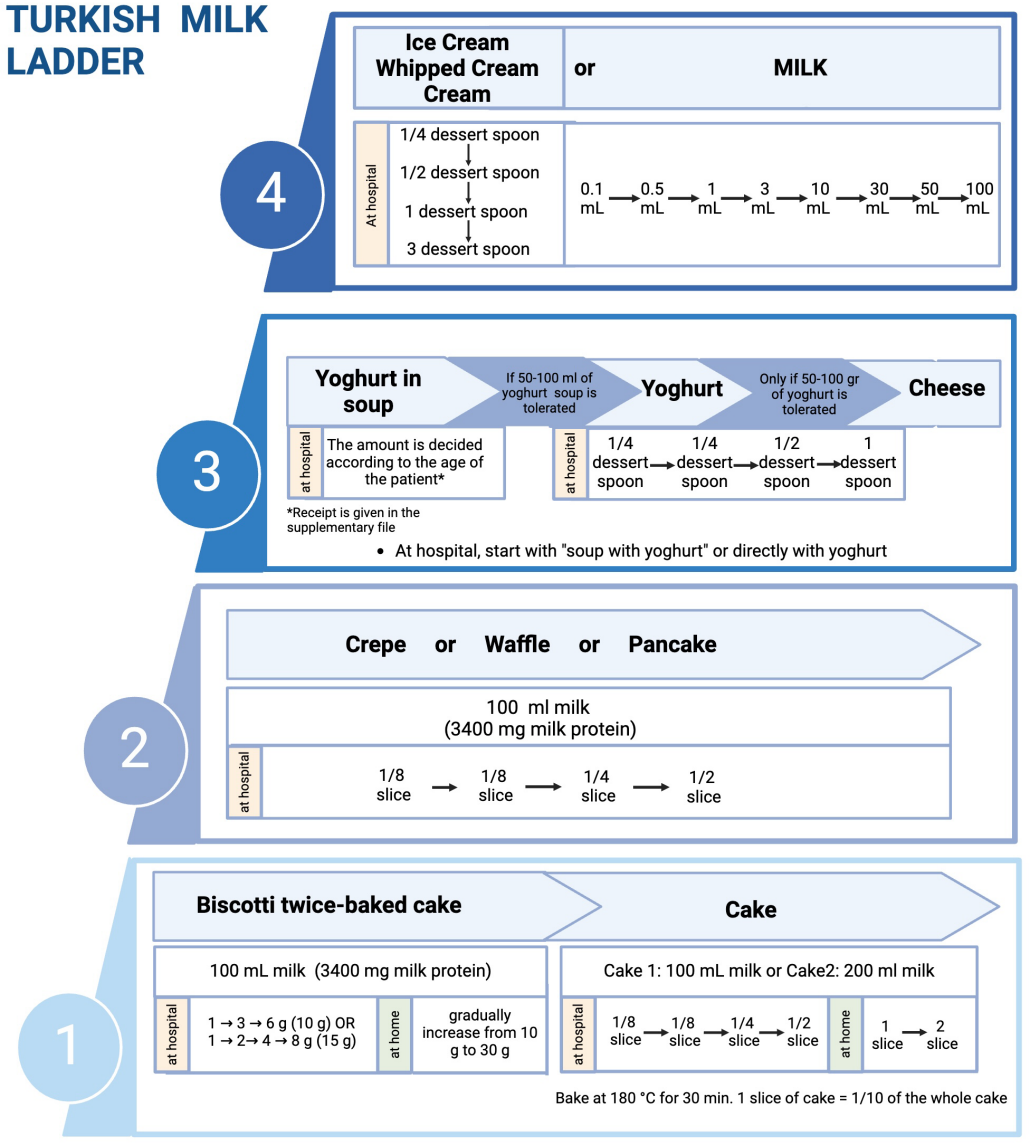
Implementation of milk ladder in clinical practice (Created in https://BioRender.com).

The authors apologize for this error and state that this does not change the scientific conclusions of the article in any way. The original article has been updated.

