# Supplementary material for: Milk ladder: Who? When? How? Where? with the lowest risk of reaction
Source: Front Allergy. 2024 Dec 6;5:1516774. doi: 10.3389/falgy.2024.1516774 (PMC11659236; doi:10.3389/falgy.2024.1516774)
Supplement: Supplementary file 1 [file Datasheet1.pdf]

Supplementary Table 1. Recipes for Step 1

Step	Step 1	Recipes of the Step-1 products	
Baking degree	- Baking at 180°C	1 teaspoon: 5 ml<br>1 dessert spoon: 10 ml<br>1 tablespoon: 20 ml<br>1 glass: 200 ml	
Duration of baking	- At least 30 min		
Preparation with	- Flour, Sugar, oil		
	**STEP 1A. Biscotti twice-baked cake**<br>AT HOSPITAL: The first challenge must be done at the hospital<br>1 g→ 3 g→ 6 g (total 10 g) or<br>1 g→2 g→ 4 g→ 8 g (total 15 g)<br>AT HOME: The amounts can be gradually increased to 30 g	**Biscotti twice-baked cake with 100 ml milk**<br>**Ingredients:**<br>- 250 g flour<br>- ½ glass (100 ml) milk (contains 3400 mg milk protein)<br>- 125 g sugar<br>- 1 packet baking powder (10 g)<br>- 60 ml vegetable oil	**Preparation:** Combine the ingredients to form a conventional dough for a milk cake. Chill the dough overnight at -20°C. The following day, bake the mixture for 30 minutes at 180°C. It is then let to cool and then cut into slices. The slices are subjected to a second baking procedure maintained at a low temperature between 90-120°C for a period of 3 hours. Grind the product in a mortar to create a powdered version of biscotti that is easier to measure in tiny quantities<br>(P.S. 1 g includes 8.5 mg milk protein)
For children <2 years<br>Begin with STEP 1 (Cake with 100 ml milk), when the patient tolerates **1 slice of cake**, proceed to the next product which contains more milk/yogurt	**STEP 1B: CAKE/MUFFIN 1**<br>↓<br>AT HOSPITAL: The first challenge must be done at the hospital<br>1/4 slice →1/4 slice→1/2 slice<br>AT HOME: 1 slice can be gradually increased to 2 slices	**Cake/Muffin 1: Baked cake with 100 ml milk**<br>**Ingredients:**<br>- 2 eggs (in case of egg allergy, do not use egg)<br>- 1/2 glass (100 ml) milk (contains 3400 mg milk protein).<br>- ¼ glass (50 ml) of vegetable oil<br>- ½ glass of granulated sugar*<br>- 1 packet baking powder (10 g)<br>- 1.5 glasses of wheat flour/whole wheat flour	**Preparation:**<br>First, whisk egg and sugar, then mix the other ingredients, pour into the cake mold (rectangular-shaped cake mold), and bake in the oven at 180°C for 30 minutes. The cake is divided into 10 equal slices. 1 slice of cake (1/10 of whole cake) contains 10 ml milk and 340 mg milk protein.<br>(P.S. This recipe can also be used for preparing 10 muffins, and 1 muffin can be used for the challenge)
For children >2-3 years<br>Begin with STEP 1 (Cake with 100 ml milk), when the patient tolerates **1-2 slices of cake**, proceed to the next product which contains more milk/yogurt	**STEP 1C: CAKE/MUFFIN 2**<br>↓<br>AT HOSPİTAL: The first challenge must be done at the hospital<br>1/4 slice → 1/4 slice→ 1/2 slice<br>AT HOME: 1 slice can be gradually increased to 2 slices	**Cake/Muffin 2: Baked cake with 200 ml milk**<br>**Ingredients:**<br>- 3 eggs (in case of egg allergy, do not use egg)<br>- 1 glass (200 ml) of milk (contains 6800 mg milk protein).<br>- 1/2 glass (100 ml) of vegetable oil<br>- 1 glass of granulated sugar*<br>- 1 packet baking powder (10 g)<br>- 2.5 glasses of wheat flour/whole wheat flour<br>- 1 packet vanilla (optional)<br>**To decrease the amount of sugar, dried fruits can be preferred*	**Preparation:**<br>First, whisk egg and sugar, then mix the other ingredients, pour into the cake mold (rectangular-shaped cake mold), and bake in the oven at 180°C for 30 minutes. The cake is divided into 10 equal slices. 1 slice of cake (1/10 of whole cake) contains 20 ml milk and 680 mg milk protein.<br>(P.S. This recipe can also be used for preparing 10 muffins, and 1 muffin can be used for the challenge)

Supplementary Table 2. Recipes for Step 2

**Step**	**Step 2**	**Recipes of the Step-2 products**	
Duration of cooking	3-5 min	1 teaspoon: 5 ml<br>1 dessert spoon: 10 ml<br>1 tablespoon: 20 ml<br>1 glass: 200 ml	
Preparation with	Flour, Sugar		
Examples:<br><br>Begin with Pancake or Crepe or Waffle	**STEP 2A: PANCAKE**<br><br>AT HOSPITAL: The first challenge must be done at the hospital<br>1/4 pancake →1/4 pancake→1/2 pancake<br><br>AT HOME: 1 pancake can be gradually increased to 2 pancakes.	**Pancake recipe (8 pancakes)**<br>Ingredients:<br>- 1 egg<br>- 1 tablespoon sugar<br>- 1/2 glass (100 ml) of milk<br>- ¾ glass of wheat flour/whole wheat flour<br>- 1/2 packet baking powder (5 g)<br>- 1/2 packet of vanilla (optional)	**Preparation**: First, whisk the eggs and sugar in a mixing bowl. Then add milk, flour, baking powder, and vanilla and continue whisking. Prepare dough that has a denser consistency than the cake. Pour little oil into the pan and distribute it all over. Pour a large spoonful of dough and wait for the dough to spread on its own. Turn the pancake over with a spatula when it has become golden and cook the other side. Take the pancakes, which are cooked on both sides on a serving plate. (1 pancake= 0.42 mg milk protein)
	**STEP 2B: CREPE**<br><br>AT HOSPİTAL: The first challenge must be done at the hospital<br>1/4 crepe →1/4 crepe→1/2 crepe<br><br>AT HOME: 1 crepe can be gradually increased to 2 crepes	**Crepe Recipe (4 crepes)**<br>Ingredients:<br>- 1 egg<br>- 1 tablespoon sugar<br>- 1/2 glass (60 g) wheat/whole wheat flour<br>- 1 dessert spoon sugar (optional)<br>- Then add: 1/2 glass (100 ml) of milk	**Preparation:** Whisk the flour, eggs, and sugar, then add milk and whisk some more. Prepare dough that is softer than the cake dough. Pour 1 teaspoon of oil into the pan and spread. Pour 1 ladle of crepe dough into the hot pan and spread it by tilting to the left and right. Turn both sides and cook on medium heat until lightly browned. (1 crepe= 0.85 mg milk protein)

Supplementary Table 3. Recipes for Step 3

Step	Step 3	Recipes of the Step-3 products	
Preparation by	- Cooking at 70-100°C<br>- Fermentation	1 teaspoon: 5 ml<br>1 dessert spoon: 10 ml<br>1 tablespoon: 20 ml<br>1 glass: 200 ml	
Duration of boiling	10 min		
Examples:<br><br>Begin with the soup including yogurt, when the patient tolerates 50-100 ml soup with yogurt, proceed to the yogurt or cheese step. Select cheese with less milk protein like labneh and fresh feta cheese.	**STEP-3A: Soup with yogurt (Yayla soup)** ↓	**Soup with yogurt (Yayla soup) Recipe:**<br>İngredients:<br>- 2.5 glasses of yogurt<br>- 2 tablespoons of wheat flour/whole wheat flour<br>- 1/3 glass of rice (cracked rice, cracked wheat or split, cracked corn can be used instead of rice, depending on the region)<br>- 1 egg (do not add in case of egg allergy)<br>- 2 glasses of water (broth can be preferred) + water in order to boil 1 glass of rice (or alternative)<br>- Salt<br>- Mint (optional)	**Preparation:** Yoghurt, flour, and egg are whisked well, cooked on the stove by mixing and boiled for a few minutes (flour smell comes out). At the same time, rice is cooked with 1 glass of water and added to the mixture. Finally, 2 glasses of water/broth and salt are added and boiled for 10 minutes. Add mint before serving and mix it (if you wish, mint is slightly heated in butter and added on top). The soup may thicken as it waits, in which case additional water can be added.
	AT HOSPİTAL: The first challenge must be done at the hospital<br>1 ml →3 ml→10 ml→20 ml		
	AT HOME: Increase gradually the amount		
	**STEP-3B: Yogurt** ↓		
	AT HOSPİTAL: The first challenge must be done at the hospital<br>1/4 dessert spoon (2.5 ml) →1/2 dessert spoon (5ml) →1 dessert spoon (10 ml)		
	AT HOME: If the patient tolerates 50-100 ml soup with yogurt, the patient may begin at home with ¼ dessert spoon→1/2 dessert spoon →1 dessert spoon		
	**STEP-3C: Cheese (labneh, fresh feta cheese)** ↓		
	AT HOSPİTAL: The first challenge must be done at the hospital<br>¼ dessert spoon→1/2 dessert spoon →1 dessert spoon		
	AT HOME: If the patient tolerates 50-100 ml soup with yogurt, the patient may begin labneh cheese at home with 1/4 dessert spoon→1/2 dessert spoon →1 dessert spoon		

Supplementary Table 4. Recipes for Step 4

**Step**	**Step 4**	
Preparation by	Pasteurized milk by freezing or pasteurized milk	1 teaspoon: 5 ml<br>1 dessert spoon: 10 ml<br>1 tablespoon: 20 ml<br>1 glass: 200 ml
Examples:	**ICE CREAM containing milk**	
Begin with one of the items depending on the patient/parents' choice	AT HOSPITAL: The first challenge must be done at the hospital<br>1/4 dessert spoon→1/2 dessert spoon →1 dessert spoon →3 dessert spoon	
	**CREAM**<br>**WHIPPED CREAM**	
	AT HOSPITAL: The first challenge must be done at the hospital<br>¼ dessert spoon→1/2 dessert spoon →1 dessert spoon →3 dessert spoon	
	**MILK**	
	AT HOSPITAL: The first challenge must be done at the hospital<br>0.1 ml→0.5 ml→1 ml→3 ml→10 ml→30 ml→50 ml→100 ml	
